# Diversification rates in Ctenodactylidae (Rodentia, Mammalia) from Mongolia

**DOI:** 10.1007/s12549-016-0265-9

**Published:** 2017-01-30

**Authors:** Adriana Oliver, Oscar Sanisidro, Bayarmaa Baatarjav, Ichinnorov Niiden, Gudrun Daxner-Höck

**Affiliations:** 1 Museo Nacional de Ciencias Naturales, MNCN-CSIC, C/José Gutiérrez Abascal, 2, 28006 Madrid, Spain; 2 Geological-Paleontological Department, Natural History Museum Vienna, Burgring 7, 1010 Vienna, Austria; 3 Institute of Paleontology and Geology, Mongolian Academy of Sciences, S. Danzan street-3/1, Ulan Bator, 15160 P.O. Box 46/650, Mongolia

**Keywords:** Asia, Rodents, Oligocene, Miocene, Cenozoic

## Abstract

Gundis, or comb rats, are rodents of the family Ctenodactylidae. Extant gundis are restricted to Africa and represent a vestige of the diversity that the ctenodactylids attained at both palaeoecological and palaeobiogeographical levels. Here, we present an updated review of the Ctenodactylidae from the Valley of Lakes, Mongolia, based on the study of large collections now available. We have recognised 13 valid species of ctenodactylids grouped into five genera: *Karakoromys*, *Huangomys*, *Tataromys*, *Yindirtemys*, and *Prodistylomys*. The ctenodactylids show an initial burst in diversification in the early Oligocene followed by a sequential generic extinction of *Karakoromys*, *Huangomys*, and *Tataromys*. A maximum richness peak at the late Oligocene was followed by a profound diversity crisis. *Yindirtemys*, the only surviving genus, persisted into the Miocene, joining three *Prodistylomys* species. These last representatives of the group disappeared coinciding with the late Xiejian faunal reorganisation (Mongolian biozone D).

## Introduction

**Fig. 1:**
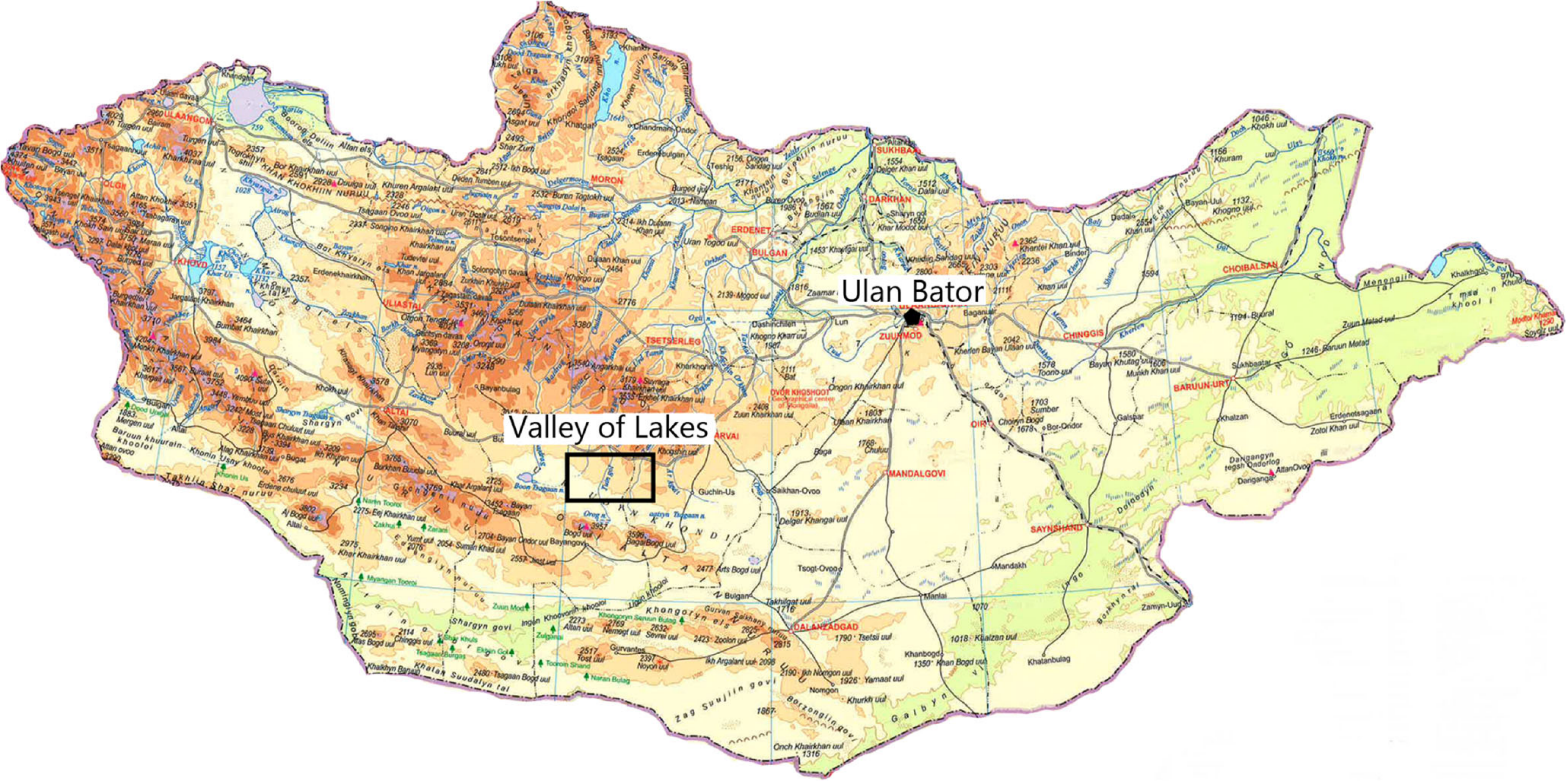
Map of Mongolia showing the location of the working area in the Valley of Lakes (modified from Oliver and Daxner-Höck in press)

The Valley of Lakes is one of the Pre-Altai depressions of Central Mongolia. It is situated within the Gobi Altai Mountains in the south and the Khangai Mountains in the north. This depression is formed by a Proterozoic to Paleozoic basement filled with terrestrial sediments ranging in age from the Cretaceous to the Quarternary. The areas of study are the Taatsiin Gol and Taatsiin Tsagaan Nuur where the exposed sediment sequence of the Hsanda Gol and Loh Fms. are very rich in Oligocene and Miocene fossils (Fig. 1). These Cenozoic sediments are interfingered with basalts. ^40^Ar/^39^Ar datings of the basalt flows define at least two groups of Oligocene basalts (basalt I, 31.5 M.a.; basalt II, 27–28 M.a.) and a middle Miocene basalt (basalt III, ∼13 M.a.; Daxner-Höck et al. 1997; Höck et al. 1999). Over eight field seasons, between 1995 and 2012, a Mongolian-Austrian team has collected in the Valley of Lakes. Eight informal local biozones have been defined according to their rodent assemblages and lithostratigraphic positions. These are A, B, C, C1, D, D1/1, D1/2, and E (Daxner-Höck and Badamgarav 2007; Daxner-Höck et al. 2013). The combination of these local mammal biozones and basalt ages provide a complete biochronology for the studied area (Daxner-Höck and Badamgarav 2007; Daxner-Höck et al. 2010, 2014, 2015, 2017).

**Fig. 2:**
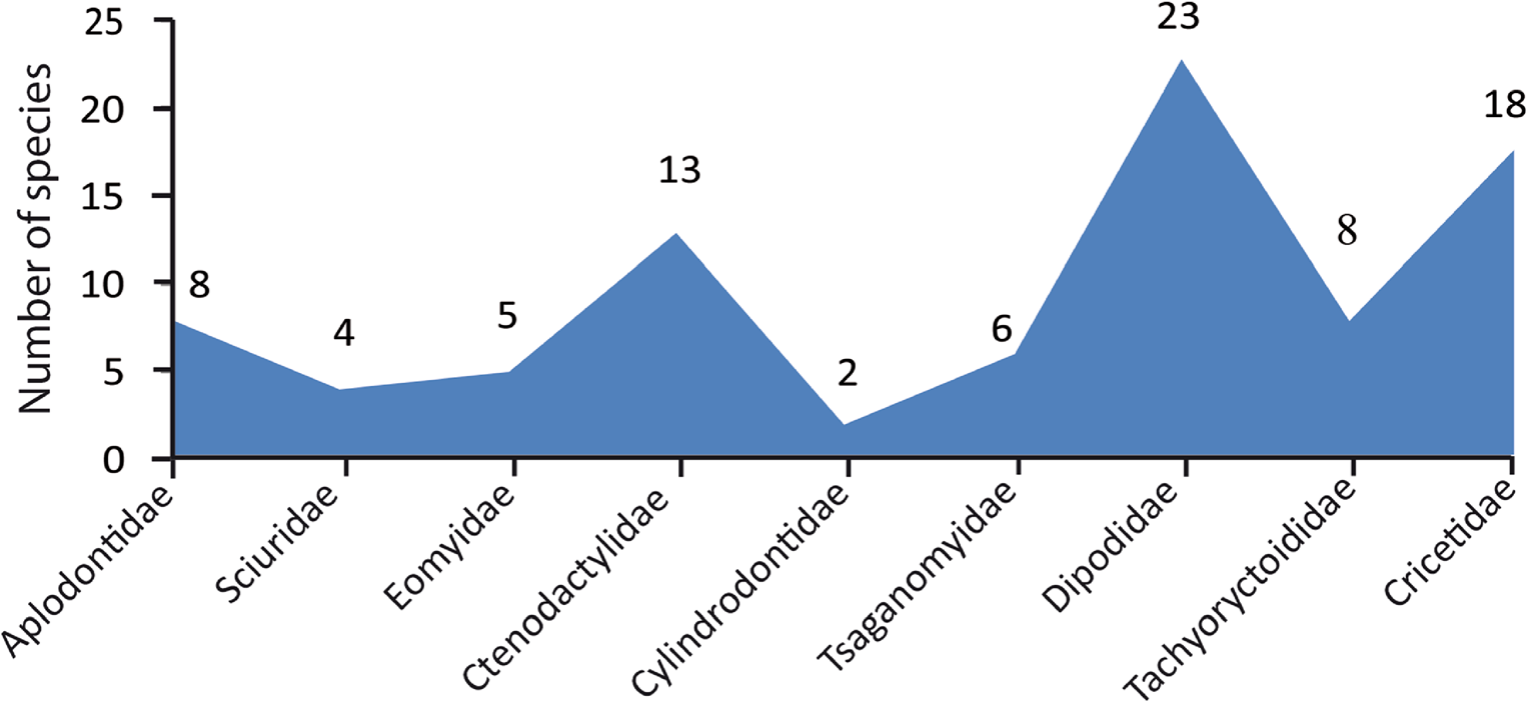
Occurrence (represented as number of species) of the different families of rodents from Mongolia during the Oligocene and early Miocene

Ctenodactylid rodents are an important part of mammal assemblages in Asia (Gomes Rodrigues et al. 2014). In the Oligocene and early Miocene of Mongolia, the family is represented by 13 species, which is 19% of the total number of rodent species (Fig. 2). The morphological characters of Ctenodactylidae are: a hystricomorphous skull and a sciurognathous mandible, well-developed lower masseteric crest and incisor enamel with multiserial microstructure and small premolars, and the upper P4 lacking metacone and hypocone. Ctenodactylidae have their first occurrence in Asia during the Paleogene. The group is divided into four subfamilies: Kakaromyinae Wang, 1994, Tataromyinae Lavocat, 1961, Distylomyidae Wang, 1994, and Ctenodactylinae Gervais, 1853. The Tataromyinae diversified and flourished during the Oligocene, spreading from East to Central Asia.

The subfamily Distylomyinae firstly occurred in the late Oligocene of China, spreading to Mongolia and diversifying in the early Miocene. Both Tataromyinae and Distylomyinae disappeared before the middle Miocene. In contrast, the subfamily Ctenodactylinae survived and diversified, reaching western Asia, Mediterranean islands, and Africa. Nowadays, Ctenodactylids are restricted to four living genera distributed along North Africa.

The fossil richness and high diversity of the group made them excellent biostratigraphic markers of the Paleogene in Asia (Bohlin 1946; Kowalski 1974; Li and Qiu 1980; Huang 1985; Wang 1997; Höck et al. 1999; Vianey-Liaud et al. 2006; Daxner-Höck and Badamgarav 2007; Daxner-Höck et al. 2010, 2015, 2017). Furthermore, they are very useful tools for palaeobiogeography and for reconstructions of palaeoclimate and paleoenvironments (Harzhauser et al. 2017).

In the last 10 years, several works of the ctenodactylids of the Valley of Lakes have been conducted (Schmidt-Kittler et al. 2006; Oliver and Daxner-Höck 2017; Oliver et al. 2016). However, these papers are mainly focused on the systematic of the different taxa, not in the overall diversity dynamics of the group. Therefore, this work updates the systematic of the Ctenodactylidae from the Valley of Lakes and emphasises the diversification trends (palaeobiostratigrapy and palaeobiogeography).

## Material and methods

**Fig. 3:**
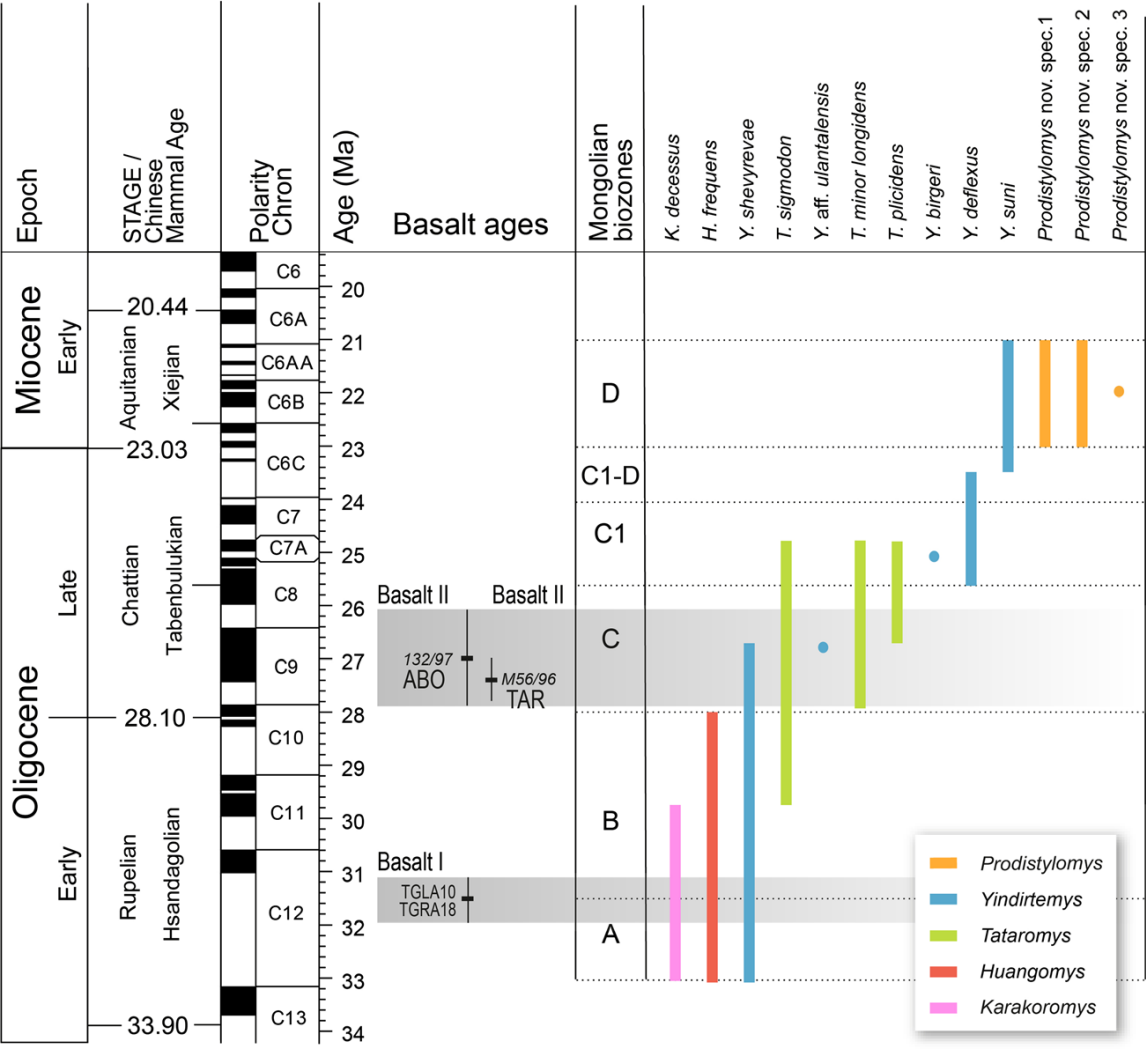
Ranges of Ctenodactylidae species from the Valley of Lakes (Mongolia). We follow the calculation of geochronologic ages of the Mongolian biozones/letter zones *A*, *B*, *C*, *C1*, *C1-D*, and *D* (Daxner-Höck et al. 2017: Figs. 30–31). The stratigraphic ranges of Ctenodactylidae species are drawn as *lines* between the first and last occurrence within the Mongolian biozones (lower or upper part of a biozone). Singleton occurrences are figured as *circles*

We constructed a dataset of Mongolian ctenodactylid occurrence data from the early Oligocene to the early Miocene of the Valley of Lakes recorded at the species level. The resulting list includes 13 species grouped in 5 genera (see Table 1). The table also includes the localities, the codes of the fossil layers/assemblages, the Mongolian biozones, and the number of specimens studied in this work. Here, we followed the calculation of geochronologic ages of the Mongolian biozones/letter zones A, B, C, C1, and C1-D (Daxner-Höck et al. 2017: Figs. 30–31). The stratigraphic ranges of Ctenodactylidae species are drawn as lines between the first and last occurrence within the Mongolian biozones (lower or upper part). The ranges do not show the number of assemblages and specimens occurring in the respective interval. Singleton occurrences are figured as circles (see Fig. 3).

**Table 1:** Distribution of the Ctenodactylidae species from the Oligocene and early Miocene of the Valley of Lakes in Mongolia

Locality	Code	Biozone	*K. decessus*	*H. frequens*	*Y. shevyrevae*	*T. sigmodon*	*T. minor longidens*	*T. plicidens*	*Y. aff.ulantatalensis*	*Y. deflexus*	*Y. birgeri*	*Y. suni*	*Prodistylomys nov spec. 1*	*Prodistylomysnov spec. 2*	*Prodistylomys nov spec. 3*
Unkheltseg	UNCH-/3+4	D										5		5	
LuugarKhudag	LOG-A/1	D												24	
HuchTeeg	RHN-A/12, −020	D												6	
HotuliinTeeg	HTE-012	D											6		
HotuliinTeeg	HTE-005+007	D													2
HotuliinTeeg	HTE-014+018	D										24			
HotuliinTeeg	HTE-003	D											2		
HotuliinTeeg	HTE-008	D										8			
HotuliinTeeg	HTE-009	D										10			
HotuliinTeeg	HTS-056/3	C1-D										1			
HotuliinTeeg	HTS-056/1-2	C1-D								18					
TatalGol	TAT-E/32	C1-D										1			
HotuliinTeeg	HTE-057	C1								35					
HotuliinTeeg	HTSE-009+013	C1				1				64					
TatalGol	TAT-E/27	C1								2					
TatalGol	TAT-E/22	C1								1					
HuchTeeg	RHN-A/23	C1								32					
HuchTeeg	RHN-A/7	C1								53					
Loh	LOH-C/1	C1								21					
Loh	LOH-B/3	C1								1					
TatalGol	TAT-W top	C1								2					
TatalGol	TAT-043	C1								18					
IkhArgalatynNuruu	IKH-B/5	C1								4					
IkhArgalatynNuruu	IKH-A/5	C1								15					
Toglorhoi	TGS	C1								1					
Toglorhoi	TGW-A surf.	C1								8					
Toglorhoi	TGW-A/5	C1								16					
TatalGol	TAT-042	C1				1				1					
TatalGol	TAT-052/1	C1								5					
TatalGol	TAT-051/2	C1								15	1				
Toglorhoi	TGW-A/3+4	C1					4	1		6					
Del	DEL-B/12	C1					21			50					
HsandaGol	SHG-AB<20top	C1						1							
TatalGol	TAT-055	C				6	2	2							
Toglorhoi	TGW-A/2a+2b	C				39	91								
UnzingChurum	TAR-A/2	C				1	14		3						
AbzagOvo	ABO-A/3	C			3		4								
TaatsiinGol (south)	TGR-C/1+2	C				4	16								
Unkheltseg	UNCH-A/3B	B		22											
HsandaGol	SHG-AB/17-20	B			9										
HsandaGol	SHG-A/15+20	B		8	3										
TaatsiinGol right	TGR-AB/22	B		43	2	1									
TaatsiinGol right	TGR-AB/21	B		60	15										
TaatsiinGol right	TGR-B/1	B		46	3										
HsandaGol	SHG-A/6-9	B	10												
TatalGol	TAT-037	A	1		2										
Khongil	HL-A/1+2	A	15												
HsandaGol	SHG-C/1-2	A	2	1											
TaatsiinGol left	TGL-A/1+2	A	4												
TaatsiinGol right	TGR-A/13+14	A	9												
TatalGol	TAT-D/1	A	11												

The table includes locality names, the codes of fossil layers/assemblages, the Mongolian biozones, the names and number of specimens of the different species. The assemblages stem from fossil layers of different sections, which were correlated with the type sections (Daxner-Höck et al. 2017: figs. 30–31)

The bulk of the material is stored in the Natural History Museum in Vienna (Austria). Additional fossil material is stored in the collection of the Institute of Palaeontology and Geology of the Mongolian Academy of Sciences in Ulan Bator (Mongolia).

The measurements have been taken using Discovery V20 and Carl Zeiss software Axiocam MRc5.

**Fig. 4:**
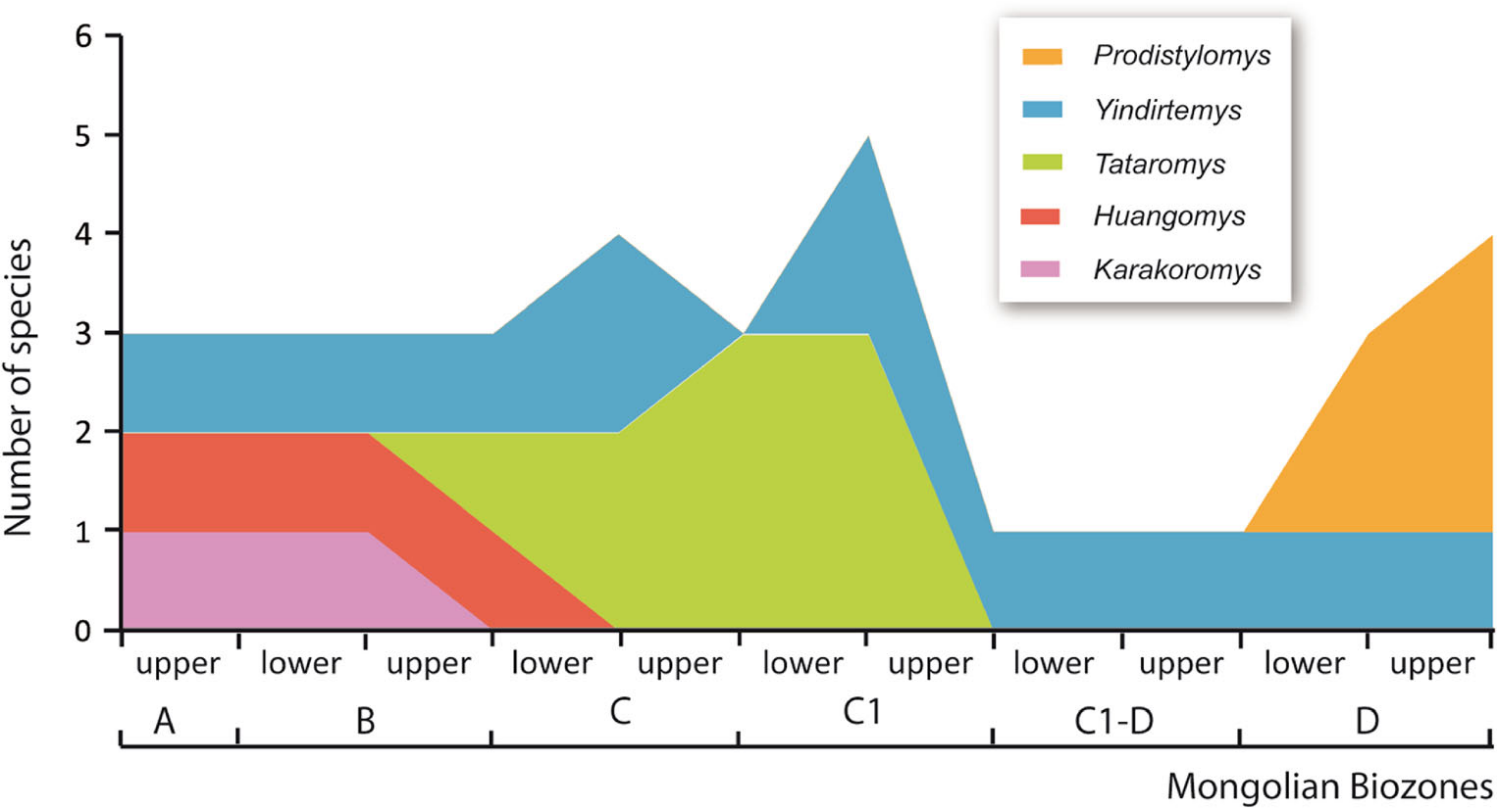
Species richness per genus through time. Richness was obtained as the sum of species’ presence in each biozone, assuming the range between first and last occurrences. The biozones are subdivided into lower and upper part

We have estimated the species richness through time using lower and upper part of the biozones. Richness was obtained as the sum of species’ presence in each biozone, assuming the range between first and last occurrences (Fig. 4).

## Systematic Palaeontology

Class Mammalia Linnaeus, 1758

Order Rodentia Bowdich, 1821

Superfamily Ctenodactyloidea Tullberg, 1899

Family Ctenodactylidae Zittel, 1893

Genus *Karakoromys* Matthew and Granger, 1923

**Type species:** *Karakoromys decessus* Matthew and Granger, 1923

*Karakoromys decessus* Matthew and Granger, 1923

**Synonymy**: *Karakoromys decessus* Matthew and Granger, 1923: 6–7, fig. 7. Bohlin 1946: 135, fig. 37. Stehlin and Schaub 1951: 288–290, fig. 494. Schaub 1958: 780, fig. 207. Mellett 1968: 6, 10. Wood 1975:125, fig. 3P. Wang et al. 1981: 28–29. Russell and Zhai 1987: 321, 329. Wang 1994: 38–40, fig. 4.Wang 1997: 49–54. Höck et al. 1999: 115–116. Vianey-Liaud et al. 2006: 121–123. Schmidt-Kittler et al. 2006: 175–180, 212. Daxner-Höck and Badamgarav 2007: 14–16. Daxner-Höck et al. 2010: 353–354, 358–359, fig. 3.1. Gomes Rodrigues et al. 2014: 7.

**Fig. 5:**
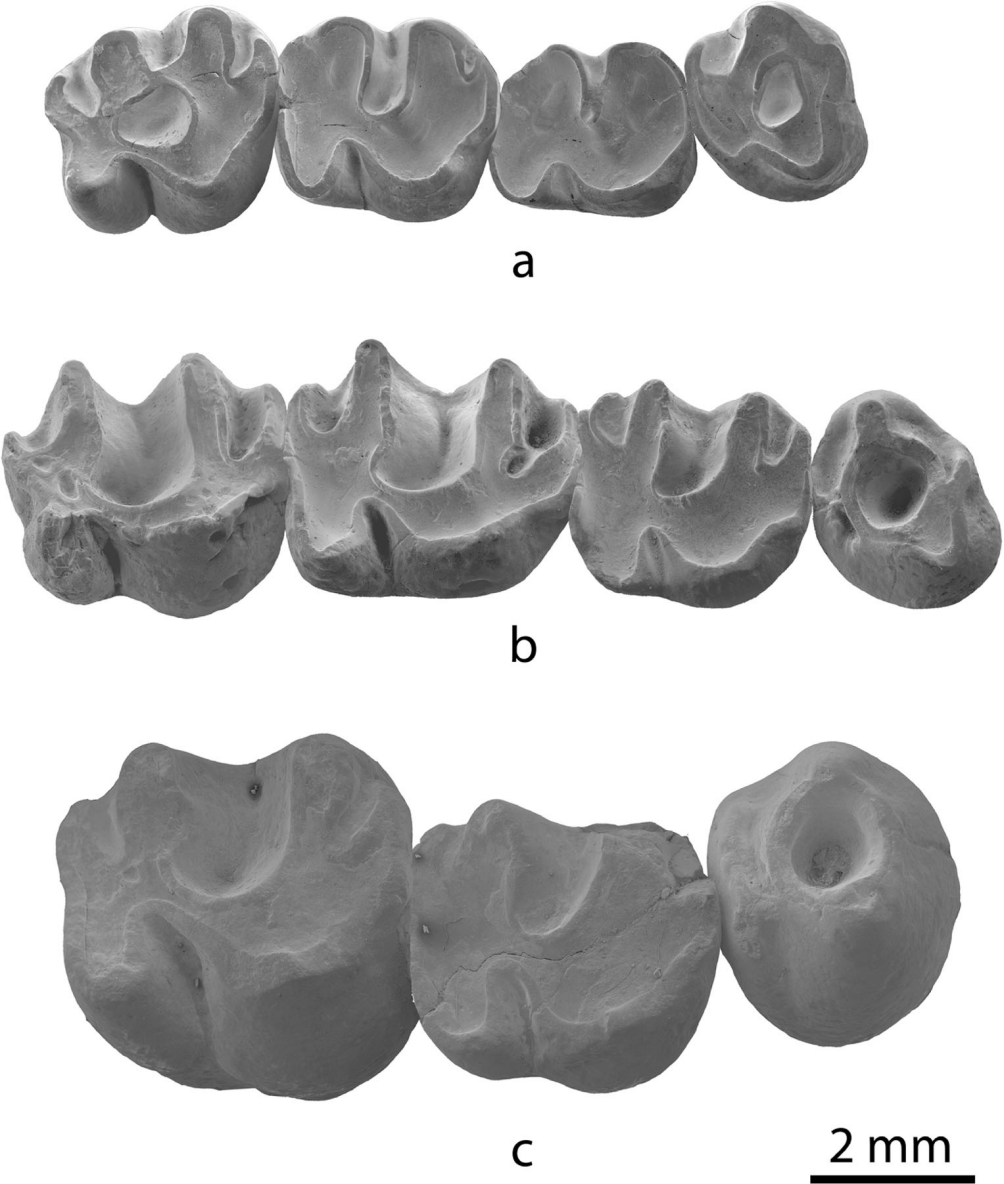
Large-sized species of *Yindirtemys* from the Valley of Lakes. **a** Right maxilla of *Yindirtemys birgeri* from Tatal Gol (TAT-051/2; NHMW 2012/0060/0001). **b** Right maxilla of *Yindirtemys deflexus* from Toglorhoi (TGW-A/surface; NHMW 2006/0086/0005). **c** Right P4-M2 of *Yindirtemys suni* from Hotuliin Teeg (HTE-014-018; NHMW 2012/0031/0001)

*Karakoromys decessus* (pro-parte): Teilhard de Chardin and Leroy, 1942: 25, 89. Kowalski 1974: 166–167, pl. XLIX, figs. 3–5, 7.

*Terrarboreus arcanus* Shevyreva, 1971:81–83, Fig. 7. Russell and Zhai 1987: 332, 345.

*Tataromys* sp. Wang et al., 1981: 28–29.

*Tataromys* spp. (pro-parte) Huang, 1982: 340–341, 347.

?*Karakoromys decessus* Huang, 1985: 36–37.

*Karakoromys decessus*? Russell and Zhai, 1987: 292, 355.

*Woodomys dimetron* Shevyreva, 1994: 116, fig. 11.

**Holotype**: Left mandible with p4-m3 AMNH19070 (1923: fig. 7).

**Type locality**: Hsanda Gol Formation, Valley of Lakes (Mongolia).

**Diagnosis** (**Vianey-Liaud et al**. 2006): Cheek teeth brachyodont; low endoloph on upper molars; metacone only weakly connected to the posteroloph; lower premolars with well developed hypoconid; lower molars and fourth deciduous premolar with ectolophid forming a protruding angle; no connection between ectolophid and metaconid.

**Stratigraphic range in Mongolia**: Early Oligocene (biozone A and lower part of biozone B); Hsanda Gol Fm. (see Fig. 3).

**Geographic distribution**: Valley of Lakes (Mongolia), Eastern Kazakhstan, Gansu province (China), Ulantatal area and Saint Jacques (Inner Mongolia, China).

**Remarks**: The material of *Karakoromys decessus* from the Valley of Lakes is relatively scarce (∼40 specimens recovered) and belongs to the three different regions: Tatal Gol in the localities of TAT-D/1 and TAT-037; Taatsiin Gol in the localities of TGR-A/13 + 14, TGL-A/1 + 2, and HL-A/1 + 2; and Hsanda Gol in the localities of SHG-C/1-2 and SHG-A/6-9 (see Table 1).

Genus *Huangomys* Schmidt-Kittler et al., 2006

**Type species:** *Huangomys frequens*

*Huangomys frequens* Schmidt-Kittler et al., 2006

**Synonymy**: *Tataromys minor* Höck et al., 1999: 117, fig. 20/7.

*Tataromyinae* nov. gen. nov. sp. Vianey-Liaud et al., 2006: 166–167, 180–181.

*Huangomys frequens* Schmidt-Kittler et al., 2006: 201–205, 214. Daxner-Höck and Badamgarav 2007: 15–16. Daxner-Höck et al. 2010: 358–360. Gomes Rodrigues et al. 2014: 7.

**Holotype**: Left mandible with p4–m3, NHMW 2006z0068/0001 (2006: 214, plate 2, fig. A).

**Type locality**: Taatsiin Gol, section TGR-B, horizon TGR-B/1, Valley of Lakes (Mongolia).

**Diagnosis**: Small ctenodactylid of the size of *Tataromys minor* with elongated molars. Upper premolar distinctly more extended in transversal direction than first upper molar. Lower premolar triangular in shape due to the lack of a hypoconid; metaconid-protoconid ridge more elongated in transversal direction than the metaconid-protoconid ridge in the first lower molar. Upper molars with well accentuated anterocone and prominent posteroloph; posterocone removed buccalwards or located at the end of the posteroloph. In the lower molars, the anterior arm of the hypoconulid bends to the lingual side and meets the entoconid; corresponding to this the hyposinusid reaches far lingually; metalophid crest-like; no trace of a trigonoid basin.

**Stratigraphic range in Mongolia**: Early Oligocene (biozone A and biozone B); Hsanda Gol Fm. (See Fig. 3).

**Geographic distribution**: Valley of Lakes (Mongolia), Ulantatal area (Inner Mongolia, China).

**Remarks**: *Huangomys frequens* occurred in two regions of the Valley of Lakes: Hsanda Gol in the localities of SHG-C/1-2 and SHG-A15-20; and Taatsiin Gol in the localities of TGR-B/1, TGR-AB/21, and TGR-AB/22 (see Table 1). This genus is one of the most common ctenodactylids from Mongolia with ∼180 specimens recovered.

Genus *Tataromys* Matthew and Granger, 1923

**Type species**: *Tataromys plicidens* Matthew and Granger, 1923

*Tataromys sigmodon* Matthew and Granger, 1923

**Synonymy**: *Tataromys sigmodon* Matthew and Granger, 1923: 6. Teilhard de Chardin and Leroy 1942: 25, 89. Mellet, 1968: 6, 10. Kowalski 1974: 164, pl. 48, fig. 4. Wang et al. 1981: 27, 29–30. Wang, 1994: 37–38, fig. 3C. Wang, 1997: 18–22, 88. Höck et al. 1999: 116. Vianey-Liaud et al. 2006: 124–132, 176–181. Schmidt-Kittler et al. 2006: 182–183. Daxner-Höck and Badamgarav 2007: 15–16. Daxner-Höck et al. 2010: 358, 360–361, fig. 5.7–5.8.Gomes Rodrigues et al. 2014: 7.

*Tataromys* cf. *plicidens* Teilhard de Chardin, 1926: 27–28, fig. 15A; pl. IV, fig. 1.

“*Karakoromys*” Bohlin, 1937: 42–43, figs. 1.01–102; pl. I, fig. 35.

*Leptotataromysgracilidens* Bohlin, 1946: 107–108, pl. II, fig. 30. Wood 1975: 125, fig. 3O.

*Tataromys* (?*Leptotataromys*) cf. *sigmodon* Stehlin and Schaub, 1951: 290–291, fig. 497.

*Tataromys* spp. (pro-parte) Huang, 1982: 340–341, 347.

**Fig. 6:**
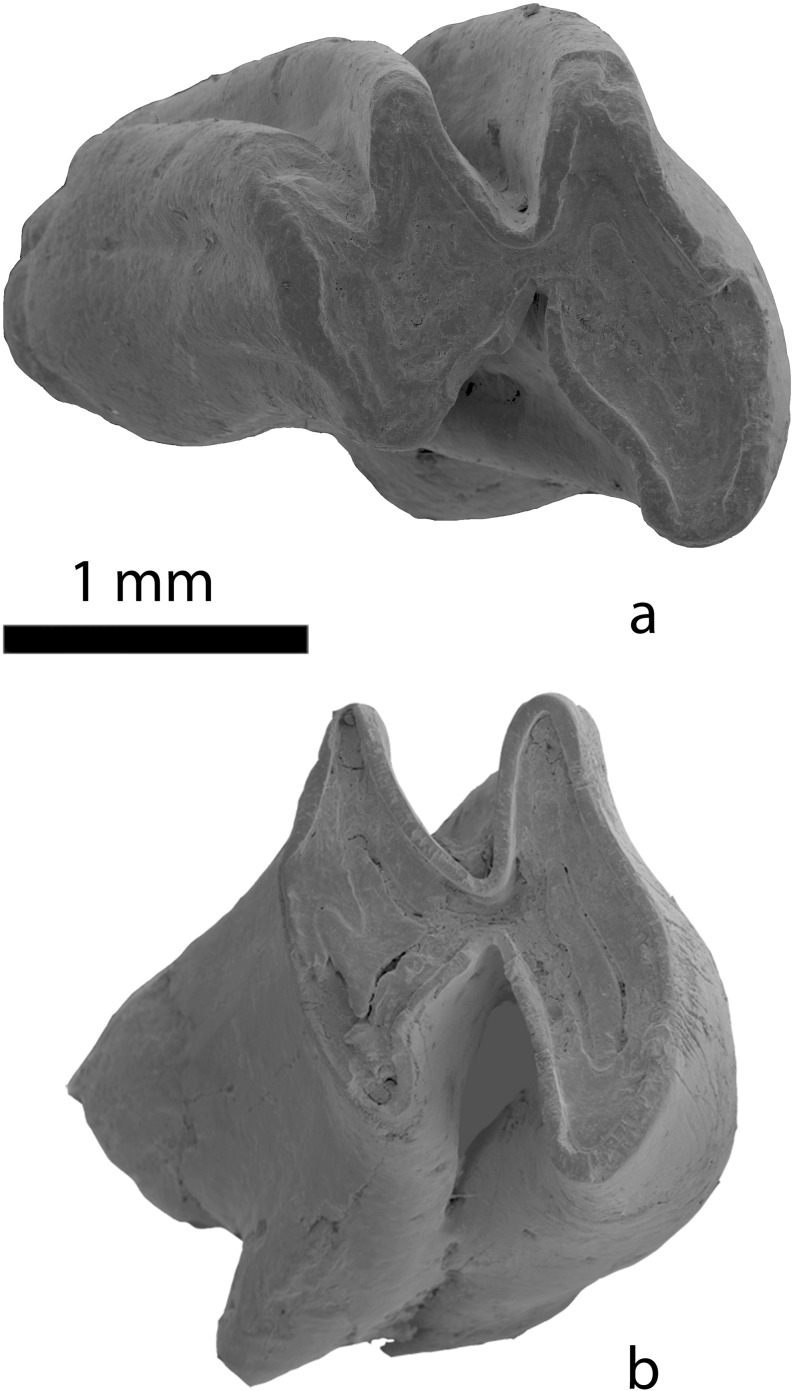
Species of *Prodistylomys* from the Valley of Lakes. **a** Right second lower molar of *Prodistylomys* nov. spec. 1 (HTE-012; NHMW 2012/0051/0001). **b** Right second lower molar of *Prodistylomys* nov. spec. 2 (LOG-A/1; NHMW 2012/0048/0001)

**Fig. 7:**
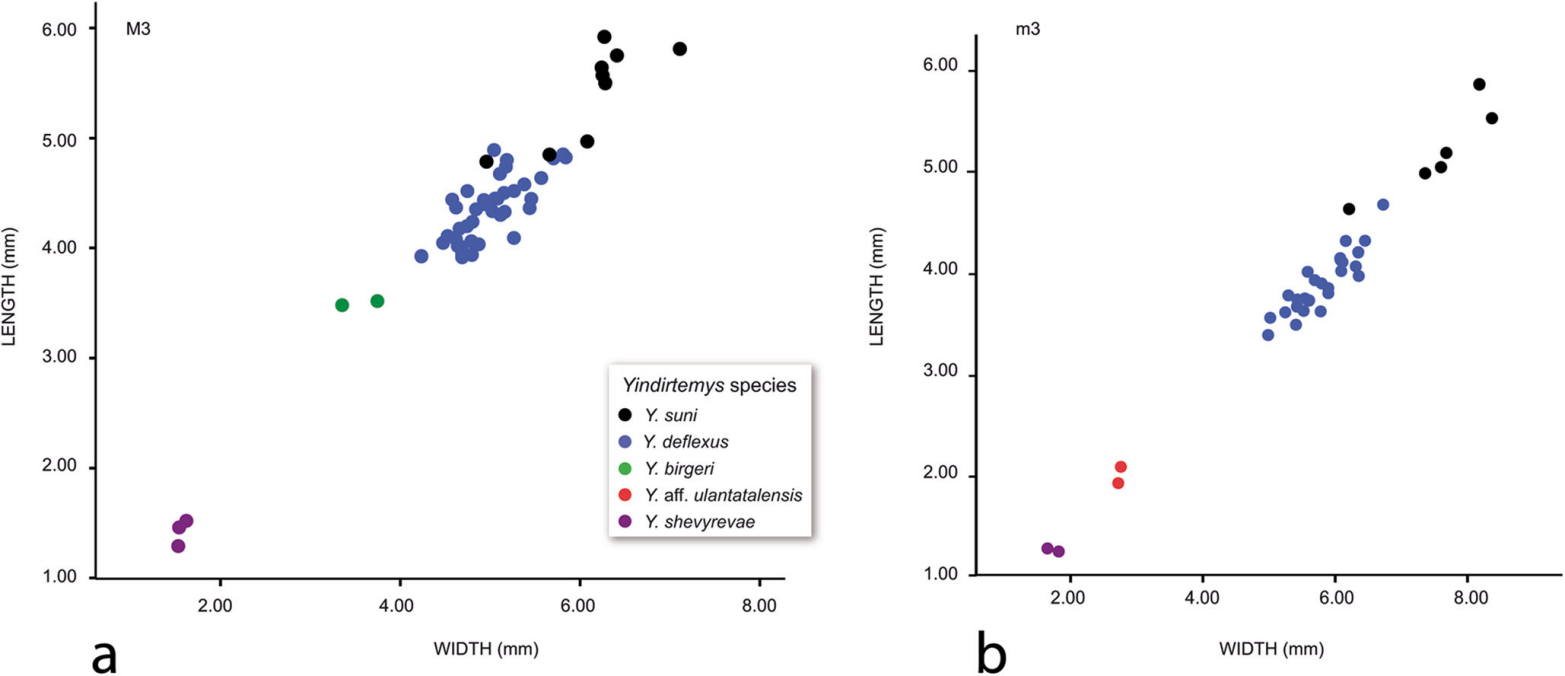
**a** Scatter diagram showing the measurements of the upper M3 of different *Yindirtemys* species from the Valley of Lakes (Mongolia). **b** Scatter diagram showing the measurements of the lower m3 of different *Yindirtemys* species from the Valley of Lakes (Mongolia)

*Leptotataromysgracilidens* (pro-parte) Huang and Wang, 1984: 39–48. Huang 1985: 32–55, 38, fig. 3; pl. II, figs. 5–9. Russell and Zhai 1987: 292, 355, 365, 395.

*Leptotataromys* cf. *gracilidens* (pro-parte) Qiu and Gu, 1988: 207, 212, pl. II, fig. 7.

*Muratkhanomys velivolus* Shevyreva, 1994: 117, figs. 1m, 2a.

*Tarromys boreas* Shevyreva, 1994: 120, figs. 2i, k.

**Holotype**: Palate with two tooth rows P4-M3 AMNH19079.

**Type locality**: Hsanda Gol Formation, Valley of Lakes (Mongolia).

**Diagnosis** (**Vianey**-**Liaud et al.** 2006): Smaller than *T. plicidens* and greater than *T. minor*; dp4/DP4 more bunodont than molars and slightly wider than P4/p4; asymmetrical P4, flattened anteriorly and rounded posteriorly; anteroloph reduced or absent, variable connections between metacone and posteroloph; general increase of length from lower p4/dp4 to m3, and from P4/DP4 or M2; great size variation of M3; on upper molars, mesosyncline L-shaped and posterosyncline short, metaloph strongly curved, reaching posteroloph on M1 and M2, posteriorly oblique and joining posteroloph on M3: morphotypes B, C, and E; on lower molars, trigonoid relatively long, usually with a relatively wide and closed basin, generally closed lingually, but sometimes superficially open on moderately worn teeth; hypoconulid usually joining arm of hypoconid; Size close to that of *Alashania tengkoliensis*; differs from *Alashania* by the shape of the dentary and the location of the foramen mentale; the development of a trigonoid basin, a short hyposinusid due to the direct junction between hypoconulid and hypoconid; metaloph joining the posteroloph (generally morphotypes B and C).

**Stratigraphic range in Mongolia**: Early Oligocene (upper part of biozone B) to late Oligocene (biozone C and lower part of biozone C1); Hsanda Gol Fm. (see Fig. 3).

**Geographic distribution**: Valley of Lakes (Mongolia), Eastern Kazakhstan, Gansu province (China), Ulantatal area (Inner Mongolia, China).

**Remarks**: The material of *Tataromys sigmodon* has been recovered in three regions: Hsanda Gol in the locality of SHG-AB > 20 top; Taatsiin Gol in the localities of TGR-AB/22, TGR-C/1 + 2, TAR-A/2, TGW-A/2a + 2b, and HTSE-009 + 013; and Tatal Gol in the localities of TAT-055 and TAT-042 (see Table 1). Approximately ∼50 specimens have been recovered.

*Tataromys minor* (Huang, 1985)

*Tataromys minor longidens* Schmidt-Kittler et al., 2006

**Synonymy**: *Karakoromys* sp. Daxner-Höck et al., 1997:

*Tataromys parvus* Höck et al., 1999: 117, fig. 20/10.

*Tataromys minor longidens* Schmidt-Kittler et al., 2006: 183–187, 212. Daxner-Höck and Badamgarav 2007: 15–16, 18. Daxner-Höck et al. 2010: 358, 360–362, fig. 5.5–5.6.

**Holotype**: Right maxilla fragment with P4 and M1, NHMW 2006z0100/0001 (Schmidt-Kittler et al., 2006: 212, pl. 1A).

**Type locality**: Loh Formation, Khunug Valley, section TGW–A/2b, Valley of Lakes (Mongolia).

**Diagnosis**: Of the size of *Tataromys minor minor* but molars more elongated, which is particularly discernable in the upper teeth; P4 larger transversally than the M1; trigonoid of the lower molars more frequently reduced.

**Stratigraphic range in Mongolia**: Late Oligocene (biozone C and lower part of biozone C1); Hsanda Gol and Loh Fms. (see Fig. 3).

**Geographic distribution**: Valley of Lakes (Mongolia).

**Remarks**: According to Schmidt-Kittler et al., 2006, this subspecies has only occurred in Mongolia, which is one of the most common ctenodactylids (∼150 specimens). The material of *Tataromys minor longidens* is from two regions: Taatsiin Gol in the localities of TGR-C/1 + 2, ABO-A/3, TAR-A/2, and TGW-A/2a + 2b; and Tatal Gol in the locality of TAT-055 (see Table 1).

*Tataromys plicidens* Matthew and Granger, 1923

**Synonymy**: *Tataromys plicidens* Matthew and Granger, 1923: 5–6, fig. 6. Stehlin and Schaub 1951: 125, fig. 179. Schaub 1958: 780–781, fig. 208. Mellett 1968: 6, 10. Kowalski 1974: 163–164, pl. 48, fig. 3. Huang 1982: 340–341, 347. Wang 1994: 37–38, figs. 2: la, lb, 3A, B, D. Wang 1997: 10–18, 87–88. Vianey-Liaud et al. 2006: 137–138, 184–185. Schmidt-Kittler et al. 2006: 181–182. Daxner-Höck and Badamgarav 2007: 15–16, 18. Daxner-Höck et al. 2010: 358. Gomes Rodrigues et al. 2014: 7.

*Tataromys* sp. Teilhard de Chardin, 1926: 28, fig. 15C.

*Karakoromys*(?) Teilhard de Chardin, 1926: 27–28, 31, fig. 15D.

?*Karakoromys decessus* (pro-parte): Teilhard de Chardin and Leroy, 1942: 25.

*Tataromys plicidens* (pro-parte): Teilhard de Chardin and Leroy, 1942: 25, 89.

?*Karakoromys* sp. Teilhard de Chardin and Leroy, 1942: 89.

*Leptotataromys gracilidens* (pro-parte): Huang and Wang, 1984: 39–48, Table 1. Huang 1985: 32–35, 38.

*Leptotataromys* cf. *gracilidens* Huang, 1985: 35, 38, pl. 2, Figs. 2–4. Russell and Zhai 1987: 292, 355.

*Muratkhanomys kulgayninae* Shevyreva, 1994: 117–119, figs. 2b.

*Roborovskia collega* Shevyreva, 1994: 120, fig. 2z, h.

**Holotype**: Right maxilla with P4-M3, AMNH 19082 (1923: Fig 6).

**Type locality**: Loh Formation, Khunug Valley, section TGW–A/2b, Valley of Lakes (Mongolia).

**Diagnosis** (**Vianey**-**Liaud et al**. 2006): The largest form known for *Tataromys*; sphenopalatine foramen above the junction of M1-M2; cheek teeth with compressed cusps and lophs; P4 anterior cingulum low; upper molars with slightly curved metaloph, weak anterocone, mesosyncline wide U-shape; anterosyncline and posterosyncline transverse; molars of morphotype A (metaloph connected to the hypocone by a short crest; variability of lower molars not well known: sometimes having very short trigonoid with or without small closed basin, or no trigonoid; hypoconulid usually joining entoconid or both entoconid and hypoconid or hypoconid on m1 and meeting hypoconid on m2 and m3.

**Stratigraphic range in Mongolia**: Late Oligocene (upper part of biozone C and lower part of biozone C1); Hsanda Gol and Loh Fms. (see Fig. 3).

**Geographic distribution**: Valley of Lakes (Mongolia), Kazakhstan, Ulantatal area, and Saint Jacques (Inner Mongolia, China).

**Remarks**: The material of *Tataromys plicidens* is very scarce in the Valley of Lakes; only four specimens have been recovered. However, the specimens belongs to three different regions: Tatal Gol (TAT-055), Hsanda Gol (SHG-AB > 20 top), and Taatsiin Gol (TGW-A/3 + 4; see Table 1).

Genus *Yindirtemys* Bohlin, 1946

Type species: *Yindirtemys grangeri* Bohlin, 1946

*Yindirtemys shevyrevae* Vianey-Liaud et al., 2006

**Synonymy**: *Yindirtemys shevyrevae* Vianey-Liaud et al., 2006: 157–164, 198–205. Schmidt-Kittler et al. 2006: 187–190, 214. Daxner-Höck and Badamgarav 2007: 15–16. Daxner-Höck et al. 2010: 358. Gomes Rodrigues et al. 2014: 7. Oliver and Daxner-Höck 2017.

**Holotype**: Fragmentary left mandible with p4-m3, UTL7-86.

**Type locality**: UTL7, from Ulantatal area, Inner Mongolia (China).

**Diagnosis**: Comparable to *Tataromys minor* and *Yindirtemys grangeri* in its size; in the lower molars, mesoconid present but less voluminous than in the species *grangeri*; no vertical groove on the posterior wall of the protoconid separating it from the mesoconid developed; cones more rounded than in *Tataromys minor* but without selonodont tendency as in *Yindirtemys grangeri*; metaloph of upper molars mostly connected to the hypocone, corresponding to pattern type B.

**Stratigraphic range in Mongolia**: Early Oligocene (biozone A and upper part of biozone B) to late Oligocene (lower part of biozone C); Hsanda Gol Fm. (see Fig. 3).

**Geographic distribution**: Valley of Lakes (Mongolia), Ulantatal area (Inner Mongolia, China).

**Remarks**: The small *Yindirtemys shevyrevae* is relatively scarce (∼40 specimens) in the Valley of Lakes. This species has occurred in three different regions: Tatal Gol in the locality of TAT-037; Taatsiin Gol in the localities of TGR-B/1, TGR-AB/21, TGR-AB/22, and ABO-A/3; and Hsanda Gol in the localities of SHG-A/15-20 and SHG-AB/17-20 (see Table 1).

*Yindirtemys* aff. ulantatalensis (Huang, 1985)

**Synonymy**: *Tataromys* spp. (pro-parte) Huang, 1982: 340–34 1, 347.

*Tataromys ulantatalensis* Huang, 1985: 28–29, fig. 1; pl. I, figs. 1–3. Russell and Zhai 1987: 292, 355.

*Leptotataromys gracilidens* (pro-parte): Huang, 1985: 32–35. Russell and Zhai 1987: 292, 355.

*Bounomys ulantatalensis*: Wang, 1994: 37–38. Wang 1997: 45–48.

*Yindirtemys ulantatalensis* Vianey-Liaud et al., 2006: 146–153, 190–195.

*Yindirtemys* aff. *ulantatalensis* Schmidt-Kittler et al., 2006: 190–191. Daxner-Höck and Badamgarav 2007: 15–16. Daxner-Höck et al. 2010: 358. Oliver and Daxner-Höck 2017.

**Holotype**: Fragment of left mandible with p4–m3, IVPP V 7341 (1985: Fig. 1 pl. 1).

**Type locality**: Upper part of the Ulantatal Formation of Ulantatal area, Inner Mongolia (China).

**Diagnosis** (**Vianey**-**Liaud et al**. 2006): Medium sized *Yindirtemys* (smaller than *Yindirtemys deflexus* and larger than *Yindirtemys bohlini*); palate nearly as wide as the molars; buno-selenodont molars, with high cusps, swollen at their bottom and acute at their top; weakly expressed and low lophs and lophids; on lower molars, high and crescentic mesoconid, at midline of the teeth; mesoconid limited by two vertical grooves, the anterior drawing a clear sinus between metaconid and mesoconid; wide trigonoid basin; additional crests present; on upper molars, anterocone high, short antesinus; clear posterosinus; morphotypes A and B most frequently observed (A: Metaloph curved forward and directly connected to the protocone; hypocone linked to the metaloph by its anterior arm; short posteroloph connected to the posterior arm of the hypocone; morphotype B: Metaloph curved backward and connected to the posteroloph-anterior arm of the hypocone junction); additional crests mainly on M3 (crochet, anti-crochet, and double junction anterocone-protocone-protoloph).

**Stratigraphic range in Mongolia:** Late Oligocene (lower part of biozone C); Hsanda Gol Fm. (see Fig. 3).

**Geographic distribution**: Valley of Lakes (Mongolia), Ulantatal area, and Saint Jacques (Inner Mongolia, China).

**Remarks**: The material of *Yindirtemys* aff. *ulantatalensis* from the Valley of Lakes has been recovered in the region of Taatsiin Gol, in the locality of TAR-A/2 (see Table 1). Schmidt-Kittler et al. (2006) assigned this scarce material (only three specimens recovered), to the species *Yindirtemys* aff. *ulantatalensis*. These authors considered that the morphological characters of the Mongolian species were identical to *Yindirtemys ulantatalensis* from Ulantatal. However, the size of the Mongolian species was within the inferior part of the size variation.

*Yindirtemys birgeri* Bendukidze, 1993.

(Fig. 5a)

**Synonymy**: *Yindirtemys birgeri* Bendukidze, 1993: 64–65, 143. Lopatin 2004: 292–293. Bendukidze et al. 2009: 351–352, 356, 368–369. Oliver and Daxner-Höck 2017.

*Yindirtemys sajakensis birgeri* Bendukidze, 1997: 207.

**Holotype**: Palate with P4–M3 left and P4–M3 right. Bendukidze (1993: pl. XXI, Fig. 2).

**Type locality**: Altyn Schokysu (Aral region, Kazakhstan).

**Emended diagnosis** (**this paper**): Medium-sized species of *Yindirtemys*; more complicated dental pattern than older *Yindirtemys*; swollen and bulky molars; upper molars with a distinct anterocone; labial anteroloph medium or long; M3 with deflexus structure; sinus transverse and deep.

**Stratigraphic range in Mongolia**: Late Oligocene (lower part of biozone C1); Hsanda Gol Fm. (see Fig. 3).

**Geographic distribution**: Valley of Lakes (Mongolia) and North Aral region (Kazakhstan).

**Remarks**: The maxilla of *Yindirtemys birgeri* from the locality of TAT-051/2, in the Tatal Gol region, is the only specimen recovered in Mongolia (see Table 1).

*Yindirtemys deflexus* (Teilhard de Chardin, 1926).

(Fig. 5b)

**Synonymy**: *Tataromys deflexus* Teilhard de Chardin, 1926: 28, 31, fig. 15B; pl. IV, fig. 3. Teilhard de Chardin and Leroy 1942: 25, 89.Stehlin and Schaub 1951: 125, fig. 181. Mellett 1968: 6, 10. Kowalski 1974: 160–161, pl. XLVII, Fig. 1. Wang et al. 1981: 29.

*Tataromys* Bohlin, 1946: 95.

*Tataromys* sp. Stehlin and Schaub, 1951: 289, fig. 496. Schaub 1958: 781, fig. 211.

*Tataromys gobiensis* Kowalski, 1974: 162.

*Yindirtemys sajakensis* Bendukidze, 1993: 60–63, pl. 20, Figs. 2–7; pl. 21. Bendukidze 1997: 207. Lopatin 2004: 298.

*Yindirtemys deflexus* Wang, 1994: 37, figs. 2a, b. Wang 1997: 30–34. Höck et al. 1999: 116–118. Vianey-Liaud et al. 2006: 164–165, 190–191. Schmidt-Kittler et al. 2006: 191–201. Daxner-Höck and Badamgarav 2007: 15–16, 18. Bendukidze et al. 2009: 351–352, 356, 368–369. Daxner-Höck et al. 2010: 358, 362–363, fig. 6.1–6.2. Gomes Rodrigues et al. 2014: 7. Oliver et al. 2016: 112. Oliver and Daxner-Höck 2017.

*Yindirtemys gobiensis* Wang, 1997: 34–35.

**Holotype**: Fragment of a right maxilla with M2 and M3. Teilhard de Chardin (1926: Fig. 15B).

**Type locality**: Saint Jacques (Inner Mongolia, China).

**Diagnosis** (**Schmidt**-**Kittler et al**. 2006): Large *Yindirtemys* species with advanced selenodonty in the lower molars and strong tendency of developing additional crests in the upper molars.

**Stratigraphic range in Mongolia**: Late Oligocene (biozone C1 and lower part of biozone C1-D); Hsanda Gol and Loh Fms. (see Fig. 3).

**Geographic distribution**: Valley of Lakes (Mongolia), North Aral region (Kazakhstan), Gansu province (China), Ulantatal area and Saint Jacques (Inner Mongolia, China).

**Remarks**: *Yindirtemys deflexus* is the most common species of ctenodactylids in Mongolia, with more than 350 specimens recovered. The material belong to four different regions: Taatsiin Gol, in the localities of DEL-B/12, TGW-A/3 + 4, TGW-A/5, TGW-A/surface, TGS, RHN-A/7 + 8, RHN-023, HTSE-009 + 013, HTE-057, and HTS-056/1-2; Tatal Gol, in the localities of TAT-051/2, TAT-052/1, TAT-042, TAT-043, TAT-W/top, TAT-E/22, and TAT-E/27; Ikh Argalatyn Nuruu, in the localities of IKH-A/5 and IKH-B/5; and Hsanda Gol, in the localities of LOH-B/3 and LOH-C/1 (see Table 1).

*Yindirtemys suni* (Li and Qiu, 1980).

(Fig. 5c)

**Synonymy**: *Tataromys suni* Li and Qiu, 1980: 205–206, 212, fig. 7; pl. I, Fig. 3. Wang et al. 1981: 27, 29, 34. Qiu and Gu 1988: 204–206, 211, pl. II, figs. 1–4, 10.

*Yindirtemys suni*: Wang, 1994: 37. Wang 1997: 35–37. Daxner-Höck and Badamgarav 2007: 16, 18. Oliver and Daxner-Höck 2017.

*Yindirtemys deflexus* (pro-parte) Schmidt-Kittler et al., 2006: 191–201.

**Holotype**: Right maxilla with P4-M3. Li and Qiu (1980: Fig. 7; pl. I, Fig. 3).

**Type locality**: Xiejia (Xining Basin, Qinghai, China).

**Diagnosis** (**Wang**, **1997**): Large species of *Yindirtemys*; upper cheek teeth having swollen cusps; P4 posterior cingulum developed; upper molars with transverse, nearly straight protoloph and metaloph, transverse mesosinus, well-developed anterocone, weakly developed antecrochet from metaloph; p4 hypoconid reduced, but always with a hypoconulid; lower molars with large open trigonid basin, round and obtuse hypoconid, and entoconid with transverse arm.

**Stratigraphic range in Mongolia**: Late Oligocene (upper part of biozone C1-D) and early Miocene (biozone D); Hsanda Gol and Loh Fms. (see Fig. 3).

**Geographic distribution**: Valley of Lakes (Mongolia), Inner Mongolia (China), and Qinghai and Gansu province (China).

**Remarks**: Schmidt-Kittler et al. (2006) assigned all large sized *Yindirtemys* specimens from Mongolia to *Y. deflexus*. However, Oliver and Daxner-Höck (2017) were able to differentiate *Y. suni* from *Y. deflexus* since new fossil material is available from several localities of the Valley of Lakes. The material of *Y. suni* from the Valley of Lakes is relatively scarce (∼50 specimens recovered) and belongs to the two regions: Tatal Gol, in the locality of TAT-E/32, and Taatsiin Gol, in the localities of HTS-056/3, HTE-009, HTE-008, HTE-014-018, and UNCH-A/3 + 4 (see Table 1).

Genus *Prodistylomys* Wang and Qi, 1989

Type species. *Prodistylomys xinjiangensis* Wang and Qi, 1989.

*Prodistylomys* nov. spec. 1 Oliver et al (in prep)

(Fig. 6b)

**Stratigraphical range**: Early Miocene (biozone D); Loh Fm. (see Fig. 3).

**Geographical range**: Valley of Lakes, Mongolia.

**Remarks**: The description of this new species will be published in a forthcoming paper. *Prodistylomys* nov. spec. 1 is the largest *Prodistylomys* in the Valley of Lakes. This species occurred in the region of Taatsiin Gol (localities of HTE-003 and HTE-012; see Table 1). The recovered material is very scarce (about 10 specimens).

*Prodistylomys* nov. spec. 2 Oliver et al (in prep)

(Fig. 6a)

**Synonymy**: *Distylomys*/*Prodistylomys* sp. Höck et al., 1999: 118. 118–119, fig. 21/1.

*Distylomys* sp. Daxner-Höck and Badamgarav, 2007: 16, 18.

**Stratigraphical range**: Early Miocene (biozone D); Loh Fm. (see Fig. 3).

**Geographical range**: Valley of Lakes, Mongolia.

**Remarks**: The description of this new species will be published in a forthcoming paper. *Prodistylomys* nov. spec. 2 is the commonest *Prodistylomys* species in the Valley of Lakes. However, in comparison with other ctenodactylids, the fossils recovered are relatively scarce (35 specimens). This mediumsized species occurred in the Taatsiin Gol region, in the localities of RHN-A/12, RHN-020, UNCH-A/3 + 4, and LOG-A/1 (see Table 1).

*Prodistylomys* nov. spec. 3 Oliver et al (in prep)

**Stratigraphical range**: Early Miocene (upper part of biozone D). Loh Fm. (see Fig. 3).

**Geographical range**: Valley of Lakes, Mongolia.

**Remarks**: The description of this species will be published in a forthcoming paper. *Prodistylomys* nov. spec. 3 is very scarce, only one specimen recorded in the locality of HTE-005 (Taatsiin Gol region).

## Diversity and size changes of the Mongolian Ctenodactylidae

Four different groups have been defined according to the morphology and size of the Mongolian ctenodactylids:

1. Ctenodactylids from the early Oligocene (biozones A and B) and early late Oligocene (biozone C) as *Kakaromys decessus*, *Huangomys frequens*, *Yindirtemys shevyrevae*, and *Tataromys minor longidens* are typically small-sized. Additionally, these species show bunodont teeth, with relatively simple dental pattern. Furthermore, height of the crown is very low.
2. Part of the early late Oligocene (biozone C) ctenodactylids such as *Yindirtemys* aff. *ulantatalensis*, *Tataromys sigmodon*, and *T. plicidens* form the second group. These species retain a comparable dental morphology, but, are larger than the previous ones.
3. In contrast to the small early Oligocene forms, the ctenodactylids from late Oligocene (biozones C1, C1-D) and early Miocene (biozone D) are medium to large-sized (Fig. 3). These species are exemplified by the genus *Yindirtemys* (*Y. birgeri*, *Y. deflexus*, and *Y. suni*), whose dentition have a more complicated dental pattern and a higher degree of selenodonty and hypsodonty (i.e. the height of the crown is larger than previous).
4. The last group is restricted to the genus *Prodistylomys* from early Miocene (biozone D) with the species *Prodistylomys* nov. spec. 1, *Prodistylomys* nov. spec. 2. and *Prodistylomys* nov. spec. 3. These forms are small to medium sized and stand out for their very simple dental pattern (bilophodont), prismatic crowns and hypsodonty.

Along the Oligocene and early Miocene, there is an increase in size among the different *Yindirtemys* species (Figs. 5 and 7). The oldest ones, *Y. shevyrevae* and *Yindirtemys* aff. *ulantatalensis*, are the smallest species, whereas the youngest, *Y. deflexus* and *Y. suni*, are the largest.

Our studies on the Ctenodactilidae from Mongolia evidence that the subfamily Tataromyinae trends towards increasing size, crown height, and more developed crests, confirming previous studies (Vianey-Liaud et al. 2006; Oliver and Daxner-Höck 2017). These modifications started in the early Oligocene (biozone A), continued in the heyday of the family in late Oligocene (biozone C), increased rapidly towards the latest Oligocene (biozone C1), and ended with the extinction of the genus in the early Miocene (biozone D).

Ctenodactylidae occurred for the first time in Central Mongolia in the early Oligocene (Mongolian biozone A). Three genera dominated this biozone, *Karakoromys*, *Huangomys*, and *Yindirtemys* (Figs. 3 and 4). During biozone B, these three genera remain constant, only *Karakoromys* disappeared during the lower part of biozone B. From biozone B onwards a relatively varied ctenodactylid assemblage persists in the region, and the genus *Tataromys* first was evidenced in the higher part of biozone B.

An important climatic disturbance took place at the beginning of the late Oligocene (biozone C; 27–28 M.a.), called the Mid-Oligocene Reorganization (Harzhauser et al. 2016), coinciding with the Oligocene Glacial Maximum. This significant climatic change forced two effects in the ctenodactylid assemblages. Firstly, the species *Huangomys frequens* (a typical component from early Oligocene) went extinct. Secondly, the stable assemblages recorded at the biozone B were replaced by short-termed changing faunas towards its maximum between 26 and 25 M.a. (late Oligocene; Fig. 4) dominated by *Yindirtemys* and *Tataromys* species. Up to five ctenodactylid species are recorded at this time in the Valley of Lakes (Fig. 3).

The richness’ curve shows that the ctenodactylids experienced a drastic diversity drop towards the boundary between biozone C1 and C1-D (Fig. 4), defined as the Late Oligocene Extinction Event (Harzhauser et al. 2016), in which both *Tataromys* species and *Y. birgeri* went extinct. The genus *Yindirtemys* acted as a transitional component, being the only representative of the highly impoverished ctenodactylid faunas at the end of the late Oligocene. While *Y. deflexus*, the only survivor species from the crisis disappeared at the Oligo-Miocene boundary, *Y. suni* not only crossed it, but persisted up to the early Miocene (biozone D) as a relict of Tataromyinae. During the early Miocene, *Y. suni* was accompanied by three species of the genus *Prodistylomys*, the only genus of Distylomyinae in the area.

## Composition of Ctenodactylidae palaeocommunities in Central Asia

We have compared the specific richness of the ctenodactylids from the Valley of Lakes to that from Ulantatal (Inner Mongolia). Both areas have well-known Oligocene deposits, as well as diverse mammal faunas (Huang 1982; Daxner-Höck et al. 2010).

In the Valley of Lakes five genera and 13 species of Ctenodactylidae are evidenced (Fig. 3), whereas from Ulantatal nine genera and 16 species were described (Gomes Rodrigues et al. 2014, table 2).

In both regions, the early Oligocene is characterised by *Karakoromys*. The species *Karakoromys deccesssus* is known from Mongolia and China, *K*. cf *decessus* only from China.

*Huangomys frequens* is also common in both areas. In Mongolia it is restricted to the early Oligocene, but in China it persisted to the late Oligocene.

The genus *Helanshania* is only present in the early Oligocene of Ulantatal.

*Yindirtemys* and *Tataromys* also occurred in the early Oligocene, and developed different species. This development goes along with size increase and successively with more complicated dental pattern. In the Valley of Lakes five *Yindirtemys* species developed, two small-sized (*Y. shevyrevae* and *Y*. aff. *ulantatalensis*), one medium-sized (*Y. birgeri*) and two large-sized (*Y. deflexus* and *Y. suni*). In Ulantatal occurred also five species, four of small size (*Y. shevyrevae*, *Y*. aff. *shevyrevae*, *Y. ulantatalensis* and *Y. bohlini*) and *Y. deflexus* of large size. Three *Tataromys* species were evidenced in Mongolia and China. *T. sigmodon* and *T. plicidens* in both areas, *T. minor* in Ulantatal and the subspecies *T. minor longidens* in the Valley of Lakes.

In 2006, Vianey-Liaud et al. described three new genera of Tataromyinae (Tataromyinae nov. gen., nov. sp. 2, Tataromyinae nov. gen., nov. sp. 3 and Tataromyinae nov. gen., nov. sp. 4) from the late Oligocene of Ulantatal (UTL 6 and UTL 8). These new genera are restricted to this area.

The Ctenodactylidae disappeared in Ulantatal at the end of the Oligocene, however, in the Valley of Lakes the family persisted up to the early Miocene with *Y. suni* and the *Prodistylomys* species (*Prodistylomys* nov. spec. 1, *Prodistylomys* nov. spec. 2. and *Prodistylomys* nov. spec. 3). Distylomyinae are registered in China (Xinjiang and Nei Mongol) from late Oligocene (*Distylomys qianlishanensis*) to early Miocene (Wang 1997), and in Mongolia (Valley of Lakes) in the early Miocene.

The distribution of the ctenodactylids suggests that no physical barriers existed between Kazakhstan, Mongolia, and northern China throughout the Oligocene and early Miocene; the differences in the palaeocommunity are derived from different ecological niches and from different environments and/or different climatic conditions (Schmidt-Kittler et al. 2006; Bendukidze et al. 2009; Gomes Rodrigues et al. 2014; Oliver and Daxner-Höck 2017).

## Conclusions

Our study shows the diversity of Mongolian ctenodactylids. The pattern of the ctenodactylids shows three phases: A first starts as diversity burst at the early Oligocene. The second phase records an increment in the faunal replacement with higher extinction that lead to a diversity maximum in biozone C1 (at ∼26–25 M.a.; late Oligocene). We assume that the observed changes of ctenodactylid compositions are linked with the climatic instability in the course of the late Oligocene, called the Late Oligocene Extinction Event (Harzhauser et al. 2016).

Finally, the third phase is characterised by taxonomically impoverished ctenodactylid faunas of the early Miocene.

The history of the Ctenodactylidae in Mongolia was influenced by palaeogeographic reorganisation (transformations of local palaeoenvironments) and by overall climate changes towards increasing aridity (see Harzhauser et al. 2016).
